# qRAT: an R-based stand-alone application for relative expression analysis of RT-qPCR data

**DOI:** 10.1186/s12859-022-04823-7

**Published:** 2022-07-19

**Authors:** Daniel Flatschacher, Verena Speckbacher, Susanne Zeilinger

**Affiliations:** Department of Microbiology, University of Innsbruck, Innsbruck, Austria

**Keywords:** Reverse transcription quantitative real-time (RT-qPCR ), Shiny, Relative quantification, Relative expression analysis, Fold change, R, DdCq, DdCt, Inter-plate calibration (IPC )

## Abstract

**Background:**

Reverse transcription quantitative real-time PCR (RT-qPCR) is a well-established method for analysing gene expression. Most RT-qPCR experiments in the field of microbiology aim for the detection of transcriptional changes by relative quantification, which means the comparison of the expression level of a specific gene between different samples by the application of a calibration condition and internal reference genes. Due to the numerous data processing procedures and factors that can influence the final result, relative expression analysis and interpretation of RT-qPCR data are still not trivial and often necessitate the use of multiple separate software packages capable of performing specific functions.

**Results:**

Here we present qRAT, a stand-alone desktop application based on R that automatically processes raw output data from any qPCR machine using well-established and state-of-the-art statistical and graphical techniques. The ability of qRAT to analyse RT-qPCR data was evaluated using two example datasets generated in our laboratory. The tool successfully completed the procedure in both cases, returning the expected results. The current implementation includes functionalities for parsing, filtering, normalizing and visualisation of relative RT-qPCR data, like the determination of the relative quantity and the fold change of differentially expressed genes as well as the correction of inter-plate variation for multiple-plate experiments.

**Conclusion:**

qRAT provides a comprehensive, straightforward, and easy-to-use solution for the relative quantification of RT-qPCR data that requires no programming knowledge or additional software installation. All application features are available for free and without requiring a login or registration.

**Supplementary Information:**

The online version contains supplementary material available at 10.1186/s12859-022-04823-7.

## Background

Reverse transcription quantitative real-time PCR (RT-qPCR) is generally viewed by researchers as a powerful technique for accurate, sensitive and fast measurement of gene expression [1]. Despite the fact that the data acquisition step in RT-qPCR experiments is already automated - with thermocycler software providing “by-default” pre-processing procedures - the analysis of RT-qPCR data via relative quantification can still be challenging [1, 2].

Relative quantification frequently comes along with a high number of samples and replicates, resulting in experimental setups spread over multiple well-plates, substantiating the necessity for internal controls. Furthermore, the high-throughput character of RT-qPCR analysis easily leads to a rapid accumulation of raw data that needs to be quality controlled and interpreted correctly in order to obtain reliable results and produce high quality publications. This process involves multiple steps, which can not only be very time consuming but also potentially introduces user-dependent variation and errors. This is aided by the fact that data processing and visualisation methods vary widely across licensed programs for RT-qPCR analysis, which implemented functions and settings are not always transparent to the user, therefore limiting reproducible research [3]. This may include methods for the removal of outliers, normalisation, or the handling of missing values (NA). Outliers, for example, should be removed in order to prevent major bias and large errors. However, it is important to be very cautious not to tune the data to ‘more pleasing’ results. There must be a good objective reason to remove specific replicates. User-friendly data analysis software is an invaluable tool within this process. Unfortunately, most freely available software tools cover only one step in the analysis pipeline, requiring researchers to use multiple data quality control tools. This again requires manual data import and/or conversion, which might again lead to inaccurate data and errors that are very challenging to troubleshoot. Briefly, once the quantification cycle (Cq) values are obtained from a RT-qPCR experiment, they can be used to quantify gene expression. There are two commonly used approaches for this: absolute quantification and relative quantification. Both methods require internal reference genes for normalization [4]. In the absolute quantification method, expression levels of the gene of interest and internal reference genes are determined using a calibration curve. The relative quantification method is based on the difference of Cq values between the reference gene and the gene of interest [5]. As a result, the proper method to analyse the RT-qPCR dataset is heavily dependent on the experimental setup and assay.

A number of open-access software packages and tools, including R packages and online applications have been proposed in recent years for RT-qPCR data analysis via relative quantification [2]. We have found that the majority of the reviewed tools and packages [2] are outdated (EasyqpcR, pyQPCR, QPCR) or not available anymore (CAmpER, dpcR). Tools that are built on top of Microsoft Excel, such as qBase or DART-PCR, involve copy/paste manipulations from the raw data files, necessitating the careful triage of results to fit the confines of the formulas and dataflow. This can lead to calculation errors that go unnoticed [6]. Recently developed tools, such as SATQPCR [7], PIPE-T [8] or Auto-qPCR [9], come with limitations regarding input formats, the ability to easily adjust plotting parameters, or the lack of comprehensive functionalities such as inter-plate calibration. R packages (HTqPCR, ddCt), which have been proposed in recent years, are well-documented and provide diverse functions and parameter options, making them an excellent choice for the analysis of RT-qPCR data. However, R packages usually do not offer a graphical user interface and need to be used from the command line, which can be challenging for users who are inexperienced with R or programming in general. Hence, users of R-based RT-qPCR analysis software packages need non-trivial programming skills. There are no stand-alone desktop applications for the analysis of RT-qPCR data freely available, which leverage the R programming language, providing all processing steps of relative gene expression analysis, up to inter-plate calibration and the visualization of data. To address these issues, we developed qRAT (**q**PCR **r**elative expression **a**nalysis **t**ool) - an R based stand-alone desktop application - to automate the processing of raw Cq output files from any qPCR machine using well established and state-of-the-art statistical and graphical techniques. The qRAT application was designed to allow researchers with no R programming experience to perform complex RT-qPCR data analysis in a fast, simple-to-use and user-friendly environment. A key feature of qRAT is the ability to import data without the need for additional file modifications, which helps to prevent errors during analysing the experimental data. R packages and modern rendering engines are leveraged to enhance the usability from data quality control to the generation of publication-ready figures.

## Implementation

### Development and structure

qRAT was implemented in the R computing environment [10] using the Shiny web application framework [11] and distributed as a stand-alone desktop application with Electron [12]. Even though Shiny mostly is used to create web applications, the server and client part of the application can also be set up locally for the analysis of data in situations where the user wishes to work offline, or to avoid sharing sensitive data with third party hosts or software. This setup allows qRAT to run on the users’ computer without the need for any server software or internet connection. Integrating qRAT into the Electron framework (which uses Chromium under the hood) for local deployment ensures the accessibility in the ever-evolving requirements of operating systems. Neither R programming skills nor a previous installation of R are required, as the setup of qRAT also includes a compatible version of R along with all prerequisite packages to run the application. The application is almost entirely R code, but, like any Shiny app, it was further extended using HTML, CSS, JavaScript and Bootstrap. Both, a list of package dependencies, as well as instructions to install and run the application are included in a detailed user guide available on the qRAT homepage (https://www.uibk.ac.at/microbiology/services/qrat/) and within the application. The application will be continuously updated to implement additional functions and to meet the evolving requirements of operating systems. Available updates and the respective changes will be announced on the qRAT homepage.

### Data input

qRAT processes *csv* or *txt* files containing raw Cq values for each well measured by a qPCR instrument and exported by its software. The files can be tab-, comma- or semicolon-delimited. Data import is carried out using the shiny fileInput function [11]. Due to its ability to recognize synonymous column names, qRAT is capable of reading export files generated by most qPCR instrument software programs. Nevertheless, the files’ structure must fulfil specific requirements, which are stated in detail in the user guide. In *Multiple Plates* mode the user can select and input multiple files at once. Example input files for single and multiple plate analysis are provided on the qRAT homepage. HTqPCR is the chosen R package that is used to read in the data. If the file format and structure are correct, qRAT populates a qPCRset object with the raw Cq values, sample names, and targets/genes using the readCtData function [13].

### Data processing and quantification

Once data is loaded, the quality of the input data will be checked. The quality control parameters include an adjustable threshold for maximum replicate variability and a range of acceptable Cq values (Cq Cut-off). The standard deviation of the technical replicates for a given sample is calculated to detect outliers. When the standard deviation value exceeds the predefined threshold, the divergent value is removed. The adjustable standard threshold is automatically set to 0.3 as suggested by the MIQE guidelines [14]. Keeping the variation between technical replicates at a minimum is a prerequisite for precise relative quantification. Steps to minimize variability are given in Taylor et al. [1] and a strategy for pooling biological samples to reduce the amount of replicates is described by Zhang and Gant [15].

The Cq Cut-off defines lower and upper boundaries of a range of acceptable Cq values. Any Cq values outside the defined range are treated as *not available*. The adjustable default range includes any meaningful Cq value, i.e. all Cq values between 5 and 35. Depending on the settings, technical replicates are averaged within one plate (Single Plate Analysis) or across all plates of the experiment (Multiple Plates Analysis).

Relative quantities are calculated with the ddCt R package, which implements an improved relative quantification method that requires no standard curve for each primer-target pair [16]. For the validity of $\Delta \Delta$Cq calculations the amplification efficiencies of all primers for the target and the reference genes have to be approximately equal and close to 1 as stated by Livak and Schmittgen [17]. Most qPCR machines include evaluation of the primer efficiency in their accompanying software. Alternatively, different companies offer freely available online tools for primer efficiency calculation. Furthermore, according to the MIQE guidelines, at least three reference genes that are stable across all treatments shall be included in the sample set to ensure the validity of data normalization [14]. If more than one reference gene is specified, the algorithm uses the arithmetic mean of the Cq values of the reference genes for normalization. In qRAT the $\Delta$Cq values are calculated by subtracting the Cq value of the reference sample from the corresponding Cq value of the target sample ($\Delta Cq = Cq(target) - Cq(reference)$) implying that larger $\Delta$Cq values indicate lower expression. To accurately reflect different biological questions being posed, qRAT also shows $-\Delta Cq$ values (representing the calculation $\Delta Cq = Cq(reference) - Cq(target)$) which represent native transcriptional changes of enhanced expression levels. Testing significance of differences in $\Delta$Cq values can be achieved by referencing them to one or multiple control samples. Both approaches are based on the framework from the limma package [18]. Results include t-statistics, *p* values and standard errors. The *p.Value* is the associated *p* value and *adj.P.Value* is the *p* value adjusted by Benjamini and Hochberg’s method to control the false discovery rate [19]. Additional *p* value adjustment methods available are Bonferroni [20] and Holm [21]. Relative quantification is expressed either as relative quantity $RQ = 2^{-\Delta Cq}$, where samples are normalized to reference gene(s) or fold change $FC = 2^{-\Delta \Delta Cq}$), where additionally a given sample is considered as a calibration condition. Literature reviews provide a good overview of the key terminology used in RT-qPCR experiments, including most commonly used terms and distinctions [1, 4].

For sample-sets with samples spread across different plates, an inter-plate calibration can be applied to normalize results between different qPCR runs. The calculation is based on a method proposed by Hellemans et al. [3]. In order to detect and remove inter-plate variation, it is necessary to include one common sample in all runs/plates per common sample-set, the so-called inter-plate calibrators (IPCs). It makes no difference which sample is applied as IPC, as long as it is the same sample on all plates being compared. Multiple replicates of the IPC samples are recommended since they produce more precise results. First the geometric mean of all IPCs is calculated. Subsequently, the Cq value of the IPCs on each plate is averaged and all IPC Cq values are scaled according to the ratio of these mean Cq values across all plates. The mean of the IPC scaling factors on each plate is an estimate of the correction factor for the respective plate. The inter-plate variation in the original data set can now be removed by dividing each Cq value on each plate by the corresponding correction factor.

### Data visualization and outputting

The interactive nature of the plots included in qRAT allows users to explore the results of their analyses. All tables are generated using the DT R package [22]. Tables include a header with data export functions, which copy the table data to the clipboard, or export the table data into multiple formats such as *pdf* or *csv*. This facilitates the transfer to supplemental data of publications or the export to programs like Microsoft Excel or Libre Office Calc. Result tables of $\Delta$Cq and $\Delta \Delta$Cq analyses contain all calculated values for each gene/sample combination, e.g. mean values of $\Delta$Cq, standard errors and relative quantity or mean values of $\Delta \Delta$Cq, standard errors and fold change, respectively. Values in tables are rounded to two digits. Plots are drawn using the ggplot2 and plotly R packages [23, 24]. Every plot is fully customisable and includes a menu in the top right corner with various control options. Plots can be exported as *svg*, *png*, *jpeg* or *webp* according to your image export settings, to easily import them into presentation programs like PowerPoint, or further modify them in vector programs like CorelDraw and Inkscape.

### RNA extraction and qPCR

To illustrate the capability of qRAT in analysing RT-qPCR data we used two different real data-sets produced in our laboratory. Untreated (control; C) and treated (treatment; T) mycelial samples (wildtype (WT) or mutant (M)) were harvested from fungal liquid cultures of two different sample sets (Multi-Plate (MP1, MP2) and Single-Plate (SP)). Three biological replicates were pooled as one representative sample and RNA was extracted with TRIzol Reagent (Invitrogen, Karlsruhe, Germany) according to the manufacturers’ protocol. Digestion with DNase I and reverse transcription of RNA was done with a 1:1 ratio of random hexamer and oligo(dT) primers applying the RevertAid H Minus First Strand cDNA Synthesis Kit (ThermoFisher Scientific Baltic UAB, Vilnius, Lithuania). Quantitative real-time PCR was performed in $\ge$ 3 technical replicates with GoTaq qPCR Master Mix (Promega Corporation, Madison, USA; sample set MP1 and MP2) or Luna Universal qPCR Master Mix (New England Biolabs GmbH, Frankfurt am Main, Deutschland; sample set SP) on qTOWER $^{3}$G cycler (Analytik Jena AG, Jena, Germany). Primer design for the reference gene (ref G) and the genes of interest (GOI) was done with primer3prefold and primer3plus (Version 2.4.2) [25]. Basic data processing (melting curve analysis, threshold settings, verification of equal primer efficiencies) was done with qPCRsoft 4.1 software (Analytik Jena AG, Jena, Germany), exported as a ‘semi-colon delimited’ *csv* file and analysed by qRAT, where data was normalized to ref G and C was set as calibrator. Data normalization was applied according to Livak [17].

## Results

**Fig. 1:**
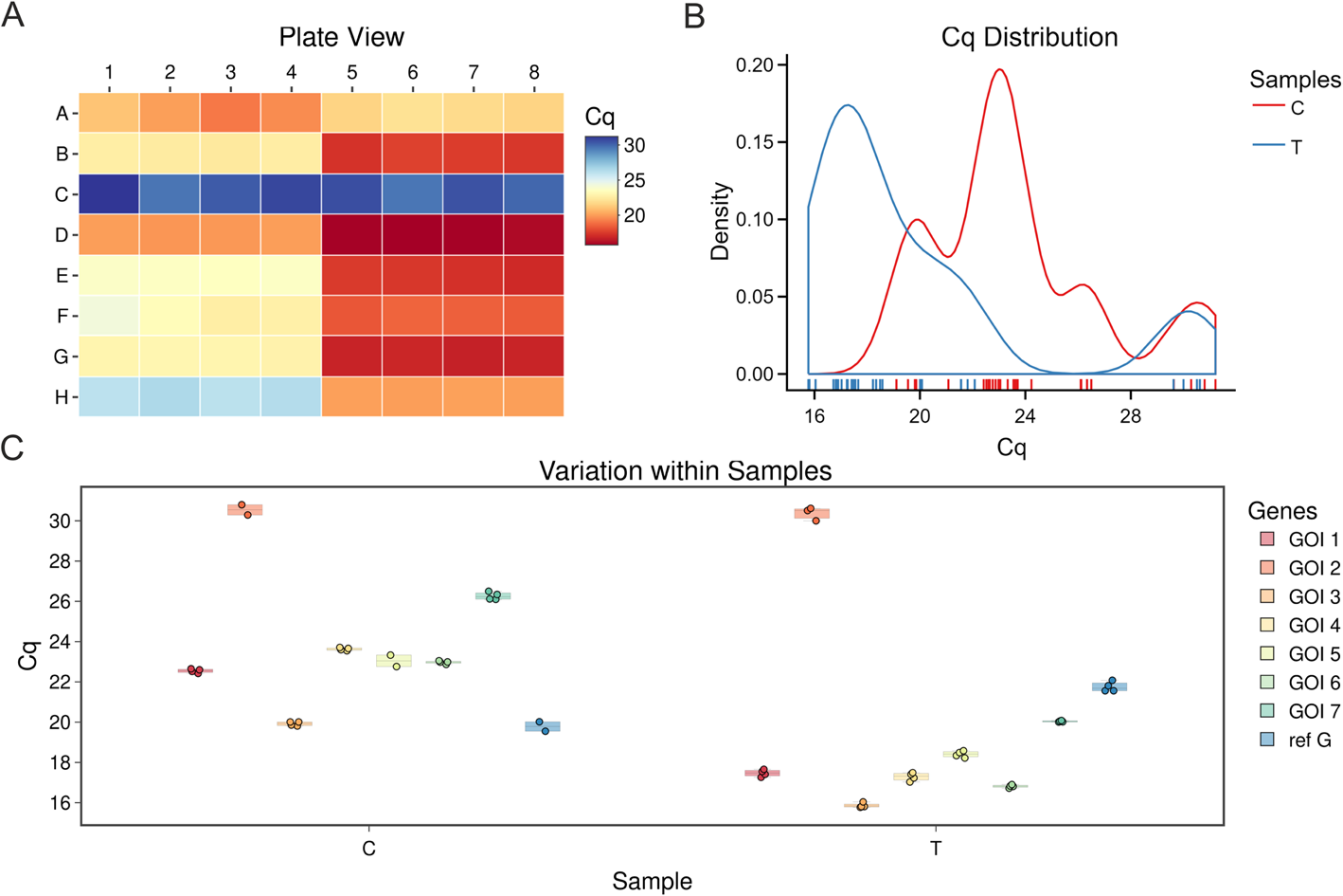
Qualitative assessment of the raw Cq values of untreated (control; C) and treated (treatment; T) samples in the SP dataset. **A** Plate view chart representing a heatmap of all Cq values. **B** Density plot of all Cq values grouped in C and T. **C** Boxplot of Cq values of C and T. The colour legend displays the target genes (gene of interest (GOI 1–7), reference gene (ref G)

**Fig. 2:**
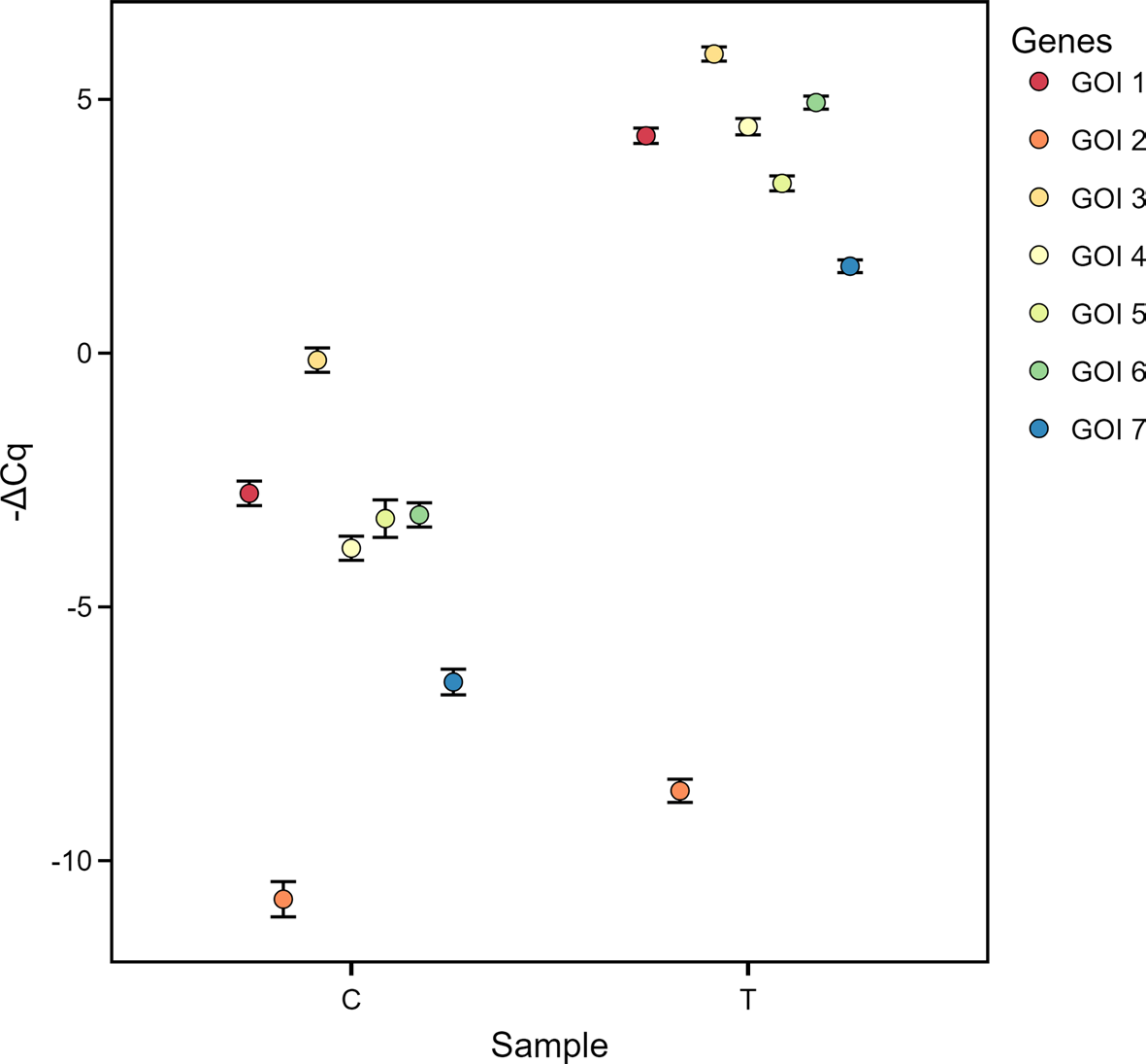
Output of qRAT using the SP dataset with the $\Delta$Cq model. Dot plot showing the $-\Delta$Cq of the genes of interest (GOI 1–7) of control sample (C) and treatment sample (T)

**Fig. 3:**
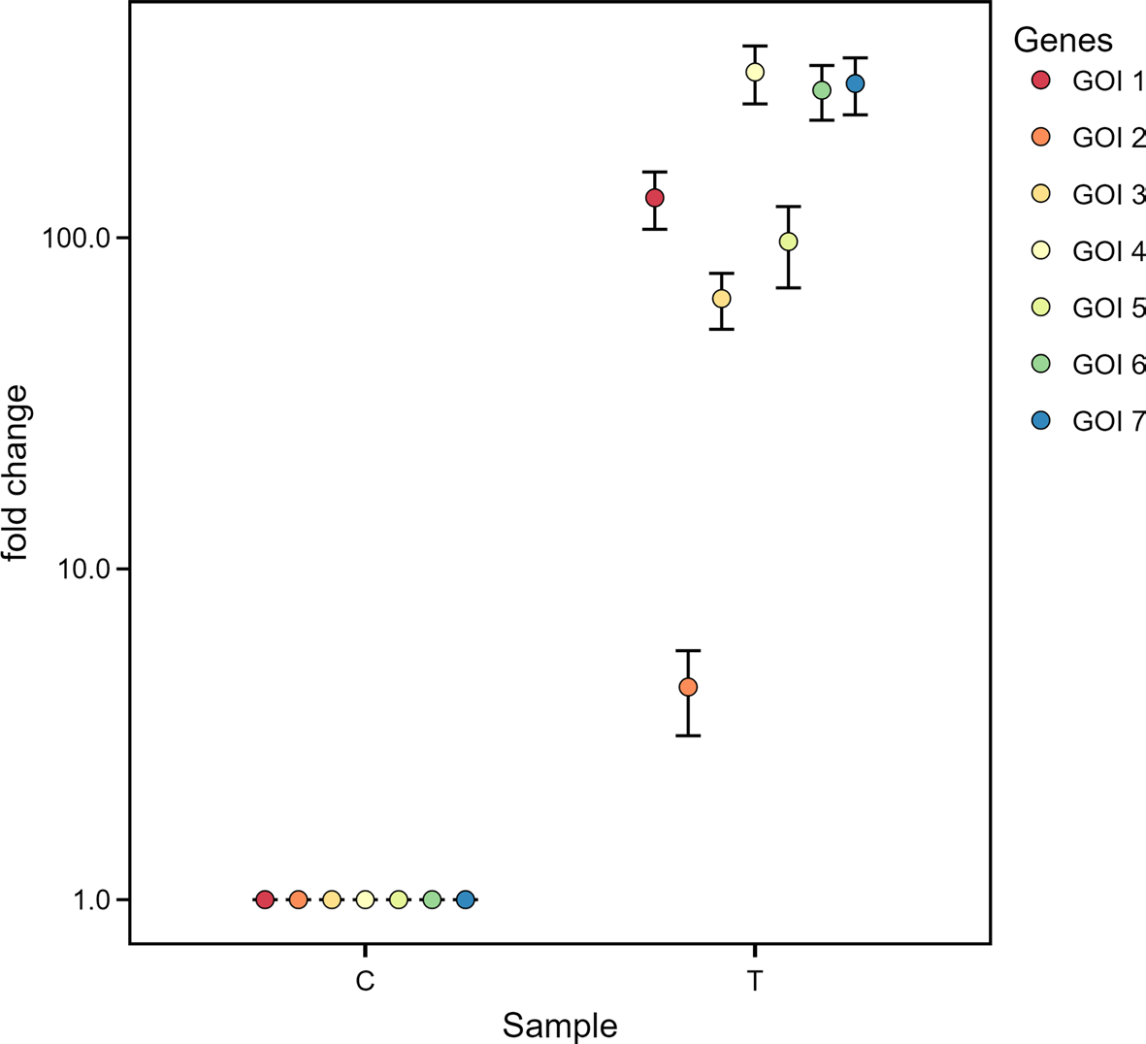
Output of qRAT using the SP dataset with the $\Delta \Delta$Cq model. Dot plot showing the fold change of the genes of interest (GOI 1–7), of control sample (C) and treatment sample (T)

The ability of qRAT to analyse pre-processed RT-qPCR data was assessed using the two example datasets (SP and MP 1–2), whose semi-colon-separated *csv* files were exported from qPCRsoft 4.1 software. Both datasets are also provided as a practical example (see Additional file 1). The application successfully completed the procedure, returning tables and visualizations of the raw input data, pre-processed data, relative quantifications and statistical tests (see Tables 1, 2, Figs. 1, 2, 3). A first overview of the raw data is depicted in Fig. 1, which visualises the distribution of outliers and homogeneity of the data. The plate view chart (Fig. 1A) is inspired by the physical design of a well-plate and provides a general overview of all samples in their spatial arrangement using the heatmap principle. To address the lack of suitable graphical representations of the plate combined with Cq values in other available tools, we implemented this chart, which helps to find spatial patterns. The Cq density plot (Fig. 1B) complements the visualisation of the distribution of Cq values across all samples. The boxplot of Cq values depicts the variation within each sample and visualises possible outliers in the data (Fig. 1C). Here we applied a Cq standard deviation threshold of 0.5 in qRAT resulting in the detection of seven outliers among the technical replicates (Additional file 2). To apply the identical data filtering settings in qPCRSoft, we excluded those seven outliers manually for further data analysis in qPCRSoft.

**Table 1:** Statistical analysis of $\Delta$Cq values from the SP dataset

Sample	Gene	t Test	*p* Value	adj.*p* value	significance
T	GOI 1	$-39.7941$	<0.0001	<0.0001	****
T	GOI 2	$-7.8294$	0.0080	0.0091	**
T	GOI 3	$-19.5527$	<0.0001	<0.0001	****
T	GOI 4	$-34.116$	<0.0001	<0.0001	****
T	GOI 5	$-75.6083$	<0.0001	<0.0001	****
T	GOI 6	$-44.1599$	<0.0001	<0.0001	****
T	GOI 7	$-41.1419$	<0.0001	<0.0001	****

The output comprises value of t statistics (t.test), statistical significance of the two-sided t-test (*p* value), *p* value adjusted by Benjamini and Hochberg’s method (adj.*p* value), and asterisks indicating level of statistical significance (significance). Control sample (C) was used as calibrator

By selecting the *Relative dCq* tab in the main panel, qRAT calculates the $\Delta$Cq values and the mean relative quantity of the expression from all samples normalized to ref G. The $\Delta$Cq approach (without applying a sample as calibrator) allows a comparison of either the $\Delta$Cq values or the relative quantity for the different genes. Figure 2 shows the $-\Delta$Cq values of all GOI, with values of the sample groups T and C normalized to the ref G. Since $\Delta$Cq values have no interpretable zero value, bar charts are a commonly applied but semi-optimal choice for visualising $\Delta$Cq values. Consequently, qRAT plots the data as dot plot but also provides the option to change the plot type to *barchart*, as shown in Additional file 3. The lack of plot customisation is an often overlooked drawback of using other tools to analyse qPCR experiments. The qRAT application addresses this issue and provides highly customisable and publication-ready charts. Here we chose to display $-\Delta$Cq since this gives positive values which represent native transcriptional changes (Fig. 2).

The qRAT application also allows the identification of the significance of differential gene expression. The *statistical analysis* tab in the main panel allows all possible pairwise comparisons of $\Delta$Cq values between samples to be made. Herein, we use t-statistics and their associated *p* values to assess the significance of the observed transcriptional changes between untreated and treated samples (Table 1). Table 1 shows that all of the 7 GOIs tested are significantly upregulated in the treated samples (*p* value <0.05).

By clicking on the *Relative ddCq* tab in the main panel, qRAT calculates $\Delta \Delta$Cq and the mean fold change of the expression from all samples of T normalized to ref G and the calibrator C. Since the relative quantity and the fold change are exponentiated values it is strongly recommended to use a dot-plot with logarithmic y-axis for proper visualisation (Fig. 3). Our tool also provides the option to change the plot type to *barchart*, as shown in Additional file 4.

A comparison of the data derived from qRAT and qPCRSoft applying the relative $\Delta$Cq and the relative $\Delta \Delta$Cq method is reported in Table 2. This comparison shows that qRAT results closely matched the manual processing of the dataset in qPCRSoft.

**Table 2:** Comparison of the data output from qRAT and qPCRSoft using the SP dataset

qRAT						qPCRSoft		
Sample	Gene	$\Delta$Cq	$-\Delta$Cq	FC	FC.sd	$-\Delta$Cq	FC	FC.sd
C	GOI 1	2.76	$-2.76$	1	0	$-2.77$	1	0
C	GOI 2	10.76	$-10.76$	1	0	$-10.77$	1	0
C	GOI 3	0.14	$-0.14$	1	0	$-0.15$	1	0
C	GOI 4	3.85	$-3.85$	1	0	$-3.86$	1	0
C	GOI 5	3.26	$-3.26$	1	0	$-3.27$	1	0
C	GOI 6	3.19	$-3.19$	1	0	$-3.2$	1	0
C	GOI 7	6.48	$-6.48$	1	0	$-6.49$	1	0
T	GOI 1	$-4.28$	4.28	132.06	26	4.27	132.38	27.93
T	GOI 2	8.62	$-8.62$	4.39	1.26	$-8.63$	4.40	1.27
T	GOI 3	$-5.89$	5.89	65.46	12.6	5.88	65.34	12.60
T	GOI 4	$-4.46$	4.46	316.82	63	4.46	318.00	70.90
T	GOI 5	$-3.34$	3.34	97.34	26.84	3.34	97.40	20.10
T	GOI 6	$-4.94$	4.94	279.17	52.42	4.93	279.25	50.72
T	GOI 7	$-1.71$	1.71	292.54	57.34	1.71	293.28	51.36

Mean values are displayed for the relative $\Delta$Cq method, where values of the treatment sample (T) and control sample (C) are normalized to the reference gene, and the relative $\Delta \Delta$Cq method, where fold change (FC) values are normalized to the reference gene and made relative to the calibrator C

**Fig. 4:**
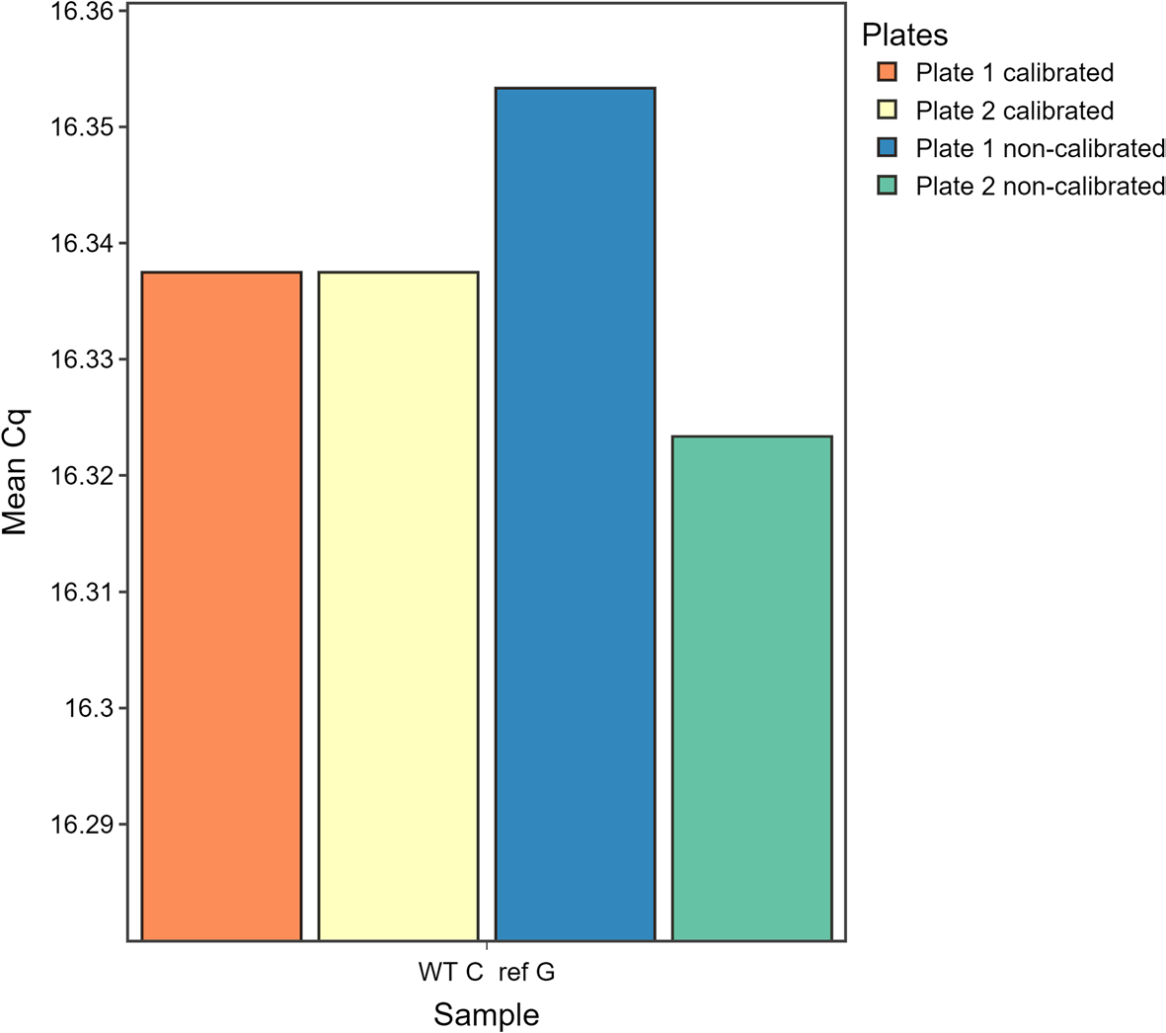
Evaluation of the impact of inter-run calibration on sample WT C ref G of the MP dataset. The difference between the Cq values is 0.02 for the non-calibrated plates, and eliminated after inter-run calibration. Orange colour indicates that data is calibrated. Blueish colour shows data before calibration. Colour shades indicate that data is derived from different runs

The MP dataset comprises samples which were measured in different runs, respectively on two different plates. Consequently, IPCs had to be included in the MP dataset. For this, qRAT offers the *Inter-Plate Calibration* function, which has to be activated in the sidebar of the *Inter-Plate Calibration* tab. Analogous to the use of three reference gene replicates for normalization, three IPC replicates were used in this dataset. Once the IPC samples have been defined, the calibration factors for each target can be found in the main panel of the *Inter-Plate Calibration* tab. Figure 4 illustrates the results of the samples WT C ref G since these samples were spread over plate 1 and 2. When comparing the non-calibrated Cq values for WT C ref G between plates 1 and 2, it was clear that those on plate 2 were higher, even though the differences between the values were already minimized in this example due to a well-designed and controlled experiment. By performing inter-plate calibration, the values on plate 1 and 2 became highly similar to each other (Fig. 4). Results of the $\Delta$Cq and the $\Delta \Delta$Cq method after inter-plate calibration are presented in Additional file 5.

In general, the sample maximization method, which states that all samples must be analysed for a given gene in the same run, should be applied whenever feasible [3]. This sample maximization strategy does not suffer from inter-plate variation, as all samples are measured in the same run for the particular gene. Therefore, inter-plate calibration is not required. In addition, there is no need for the ref G to be measured in the same plate like the GOI, since the ref G is used for normalization of the samples. The qRAT application also offers the possibility to analyse multiple plate assays following the sample maximization strategy.

The proposed tool has some limitations too, as it has not been adapted at this stage to calculate relative gene expression data while accounting for differences in primer efficiencies [26]. Moreover, it does not support grouping of samples for statistical significance testing. Thus, it is limited to test the significance of the difference between two conditions.

## Conclusion

In conclusion, qRAT is an easy-to-use R shiny application, designed for biologists with no R programming expertise to enable the straight-forward processing, analysis and visualisation of complex relative RT-qPCR data within a user-friendly environment that facilitates fast overview of their data, results and figures. Its minimal requirements make it a ready-to-use tool on every laboratory computer. The interactive interface allows users to process and explore available data quickly and switch between various plotting types, so the data is visualized and presented in a biologically meaningful and optimal, publication-ready way. In the field of microbiology, researchers often have to analyse multiple similar datasets when comparing levels of gene expression under varying environmental or experimental conditions. For example, to understand the functioning of microbes in the environment and their cellular response mechanisms, gene expression changes in the target genes that encode enzymes in metabolic or catabolic pathways are set in relation to a calibration condition, normalized to one or multiple reference gene(s), typically housekeeping gene(s). Ideally, this means that multiple gene expression studies can use the same qPCR plate layout with different primers or different samples. Using qRAT for analysis means that the user can quickly re-run an analysis on a new dataset, allowing the data produced from a given project to be calculated seamlessly, reproducibly, and correctly for multiple plates. Moreover, qRAT includes an inter-plate calibration option that allows the analysis and processing of large datasets with samples spread across various plates. The documentation provided at each step in the workflow ensures a transparent and repeatable data handling process that is less susceptible to application errors by the user. The qRAT application is scheduled to be updated regularly in the future. Functions and features not yet included (e.g., Pfaffl method, primer efficiency calculation, additional statistical tests) are envisaged to become available with forthcoming versions. The initial release version and following updates are available under a MIT license and can be downloaded from the project website (https://www.uibk.ac.at/microbiology/services/qrat/), where documentation and additional information can also be found. The source code of the application is publicly accessible at the GitHub repository (https://github.com/DaniFlat/qRAT).

## Supplementary Information

**Additional file 1.** Example datasets: A qPCR dataset containing two example files derived from both, a single plate (SP) and a multiple plate (MP1, MP2) experiment conducted in our laboratory. Datasets are semi-colon-separated *csv* files exported from qPCRsoft 4.1 software and can be used as input for qRAT.

**Additional file 2.** Output of qRAT filtering using single plate dataset: Table of detected outliers in the single plate dataset after applying a Cq standard deviation threshold of 0.5 on values of all target genes (gene of interest (GOI 1–7), reference gene (ref G)) in the treatment sample (T) and control sample (C). Respective replicate number is given in the *rp.num* column. *xlsx* file exported directly from qRAT.

**Additional file 3.** Output of qRAT using the SP dataset with the $\Delta$Cq model.: Bar chart showing the relative quantity of the genes of interest (GOI 1–7) of control sample (C) and treatment sample (T). *png* file exported directly from qRAT.

**Additional file 4.** Output of qRAT using the SP dataset with the $\Delta \Delta$Cq model: Bar chart showing the fold change of the genes of interest (GOI 1–7) of control sample (C) and treatment sample (T). *png* file exported directly from qRAT.

**Additional file 5.** Output of qRAT $\Delta$Cq and $\Delta \Delta$Cq methods using multiple plate dataset: Table of mean $\Delta$Cq, $-\Delta$Cq and relative quantity (RQ) values, where values of the mutant samples (M) and wildtype samples (WT) are normalized to the reference gene and mean $\Delta \Delta$Cq and fold change (FC) values, where M and WT samples are normalized to the reference gene and made relative to the calibrator WT C.

**Additional file 6.** Output of qRAT $\Delta$Cq and $\Delta \Delta$Cq methods using the SP dataset: Table of mean $\Delta$Cq, $-\Delta$Cq and relative quantity (RQ) values, where values of the treatment sample (T) and control sample (C) are normalized to the reference gene and mean $\Delta \Delta$Cq and fold change (FC) values, where T and C samples are normalized by both, the reference gene and the calibrator C.

## Data Availability

The software, examples and documentation are available at https://www.uibk.ac.at/microbiology/services/qrat/. The source code is provided at https://github.com/DaniFlat/qRAT. All data generated or analysed during this study are included in this published article. Project name: qRAT (qPCR relative expression analysis tool). Project home page: https://www.uibk.ac.at/microbiology/services/qrat/ Operating system(s): Windows Programming language: R. Other requirements: As the qRAT installer auto-installs R and all required R-packages, there are no additional software requirements. License: MIT. Any restrictions to use by non-academics: None.

## Abbreviations

Cq: Quantification cycle
GOI: Gene/s of interest
IPC: Inter-plate calibration
IPCs: Inter-plate calibrators
MIQE: Minimum information for publication of quantitative real-time PCR experiments
RT-qPCR: Reverse transcription quantitative real-time PCR
