# Supplementary figures and images for: qRAT: an R-based stand-alone application for relative expression analysis of RT-qPCR data
Source: BMC Bioinformatics. 2022 Jul 19;23:286. doi: 10.1186/s12859-022-04823-7 (PMC9297597; doi:10.1186/s12859-022-04823-7)

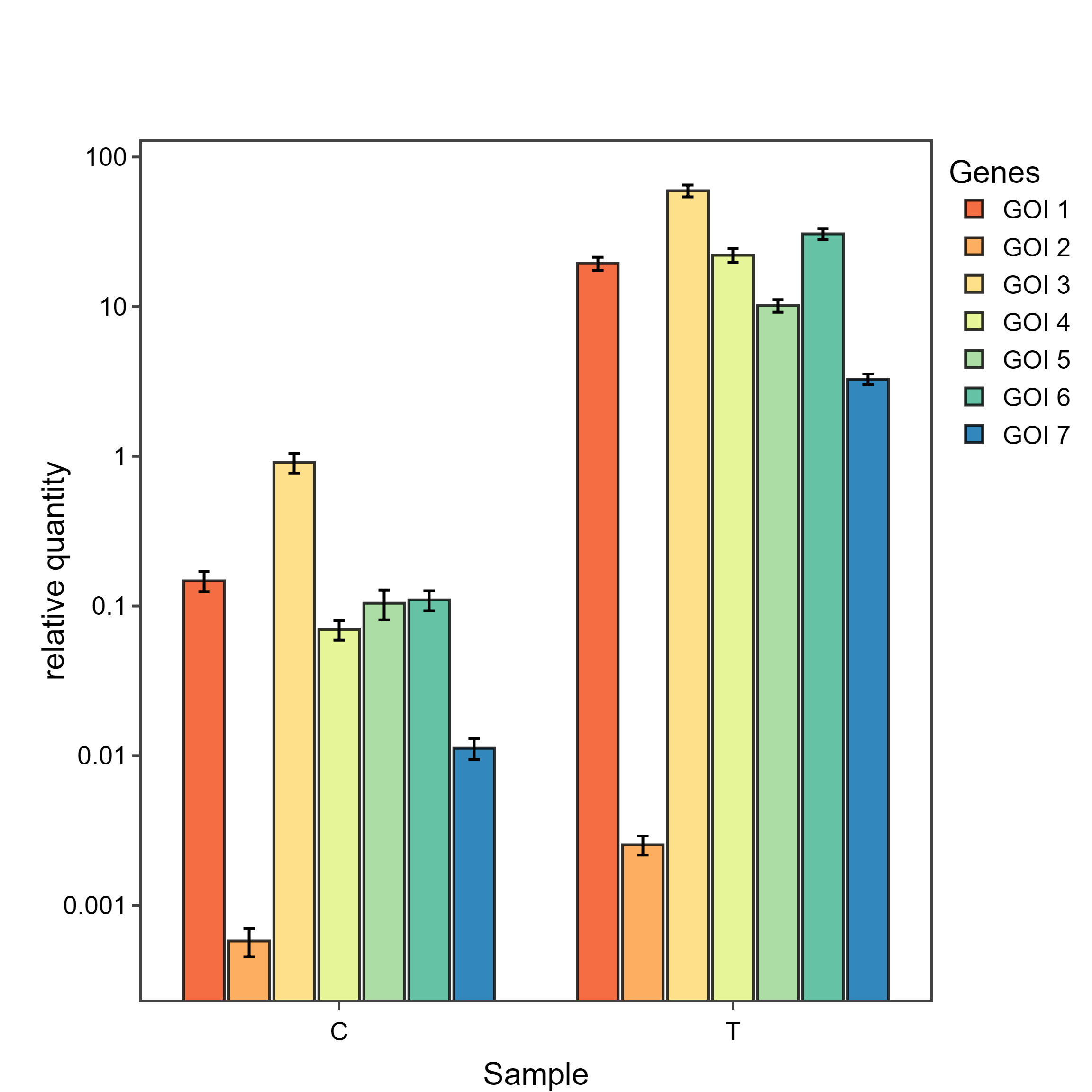

Supplement: Supplementary file 3 — Additional file 3. Output of qRAT using the SP dataset with the $\Delta$ΔCq model.: Bar chart showing the relative quantity of the genes of interest (GOI 1–7) of control sample (C) and treatment sample (T). png file exported directly from qRAT. [file 12859_2022_4823_MOESM3_ESM.png]

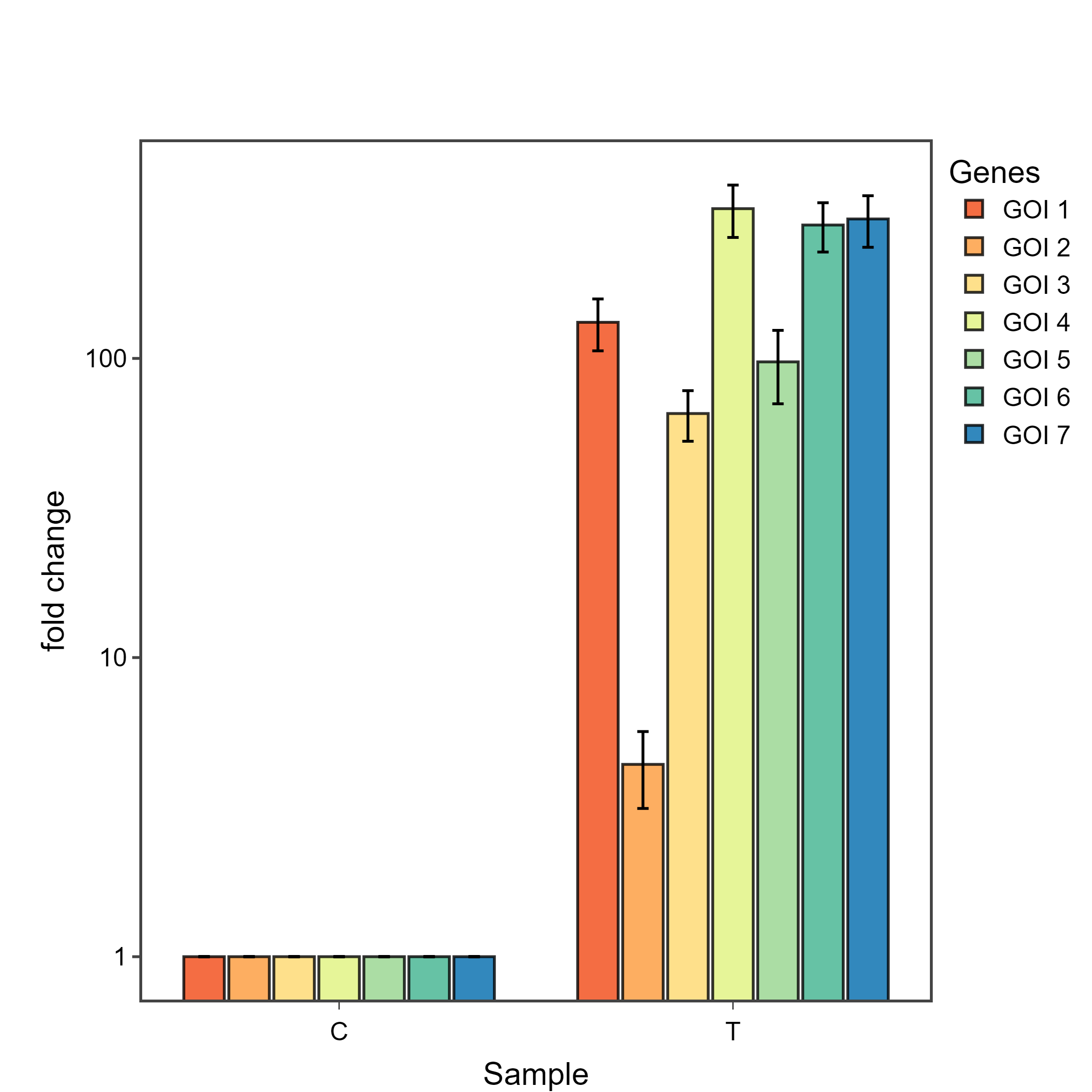

Supplement: Supplementary file 4 — Additional file 4. Output of qRAT using the SP dataset with the $\Delta \Delta$ΔΔCq model: Bar chart showing the fold change of the genes of interest (GOI 1–7) of control sample (C) and treatment sample (T). png file exported directly from qRAT. [file 12859_2022_4823_MOESM4_ESM.png]
